# Clinical and laboratory experience of vorinostat (suberoylanilide hydroxamic acid) in the treatment of cutaneous T-cell lymphoma

**DOI:** 10.1038/sj.bjc.6603465

**Published:** 2006-12-12

**Authors:** M Duvic, C Zhang

**Affiliations:** 1 Department of Dermatology, University of Texas MD Anderson Cancer Center, Box 434 1515, Holcombe Blvd, Houston, TX 77030, USA

**Keywords:** histone deacetylase inhibitor, vorinostat, suberoylanilide hydroxamic acid, SAHA, cutaneous T-cell lymphoma, mycosis fundgoides

## Abstract

The most common cutaneous T-cell lymphomas (CTCLs) – mycosis fungoides (MF) and Sézary Syndrome – are characterised by the presence of clonally expanded, skin-homing helper-memory T cells exhibiting abnormal apoptotic control mechanisms. Epigenetic modulation of genes that induce apoptosis and differentiation of malignant T cells may therefore represent an attractive new strategy for targeted therapy for T-cell lymphomas. *In vitro* studies show that vorinostat (suberoylanilide hydroxamic acid or SAHA), an oral inhibitor of class I and II histone deacetylases, induces selective apoptosis of malignant CTCL cell lines and peripheral blood lymphocytes from CTCL patients at clinically achievable doses. In a Phase IIa clinical trial, vorinostat therapy achieved a meaningful partial response (>50% reduction in disease burden) in eight out of 33 (24%) patients with heavily pretreated, advanced refractory CTCL. The most common major toxicities of oral vorinostat therapy were fatigue and gastrointestinal symptoms (diarrhoea, altered taste, nausea, and dehydration from not eating). Thrombocytopenia was dose limiting in patients receiving oral vorinostat at the higher dose induction levels of 300 mg twice daily for 14 days. These studies suggest that vorinostat represents a promising new agent in the treatment of CTCL patients. Additional studies are underway to define the exact mechanism (s) of by which vorinostat induces selective apoptosis in CTCL cells and to further evaluate the antitumour efficacy of vorinostat in a Phase IIb study in CTCL patients.

Cutaneous T-cell lymphomas (CTCLs) represent a heterogeneous group of extra-nodal non-Hodgkin lymphomas. Mycosis fungoides (MF) and its leukaemic variant, Sézary syndrome (SS), are the most frequently encountered types of CTCL. They are characterised by progressive clonal expansion of CD4+CD45RO+CLA+CCR+ helper/memory T cells that do not express the typical T-cell surface markers (CD7 and/or CD26) (Kim *et al*, 2005). In contrast to their normal counterparts, these malignant T cells exhibit abnormal apoptotic control mechanisms resulting in prolonged lifespans and are typically very resistant to treatment regimens, including chemotherapeutic agents (Meech *et al*, 2001). Epigenetic modulation of genes that induce apoptosis and differentiation of malignant T cells may therefore represent an attractive new strategy for targeted therapy of CTCL.

Recent studies in our laboratory (Zhang *et al*, 2005) indicate that vorinostat (suberoylanilide hydroxamic acid or SAHA), an oral inhibitor of class I and II histone deacetylases (HDACs), induces apoptosis of malignant CTCL cell lines and peripheral blood lymphocytes from SS patients cells at clinically achievable doses. These data suggest that alterations in HDAC activity may be involved in the pathogenesis of T-cell lymphomas and that vorinostat may be clinically effective in treating T-cell lymphomas. This article provides a brief overview of the pathophysiology of CTCL, summarises the spectrum of currently available treatment options, and finally reviews data from preclinical studies as well as ongoing or recently completed clinical trials evaluating the antitumour efficacy of vorinostat in patients with refractory or relapsed CTCL patients unresponsive to conventional therapy.

## PATHOPHYSIOLOGY AND PROGNOSIS

Mycosis fungoides represents the most common and indolent form of CTCL and was originally described more than 200 years ago (Girardi *et al*, 2004). A key characteristic of MF is the presence of malignant skin-homing T cells possessing distinct markers (CD4+CD45RO+CLA+CCR+) but lacking the usual T-cell surface markers CD7 and/or CD26. Although many clinical variants of this lymphoma are described, the most common clinical presentation is of pink to red patches or plaques appearing in sun-shielded areas. Lesions are often accompanied by variable degrees of pruritus (Girardi *et al*, 2004; Kazakov *et al*, 2004). Mycosis fungoides may progress over many years from the early patch/plaque T1–T2 stages to more advanced stages involving tumours (which frequently ulcerate), erythroderma, blood or organ involvement, and ultimately death from infection (Girardi *et al*, 2004). A leukaemic variant characterised by generalised erythroderma and the presence of abnormal circulating malignant T cells is referred to as SS. Patients with SS not only exhibit erythroderma but often have intractable itching and frequently are colonised by *Staphylococcus aureus* (Jackow *et al*, 1997). Circulating malignant lymphocytes have convoluted nuclei (referred to as Sézary cells, after the French dermatologist who discovered them) and appear on flow cytometry as CD4+CD26-lymphocytes (Jones *et al*, 2001; Washington *et al*, 2002).

The initial trigger leading to the development of skin-homing CD4+ cells in MF/SS is unclear, although environmental and infectious agents have been hypothesised. MF has an association with class II HLA DR5 and DQB^*^03 alleles, suggesting that it may be an autoimmune disease triggered by antigen presentation (Jackow *et al*, 1996). Also supporting this hypothesis is the pathologic finding of atypical, malignant epidermotropic CD4+lymphocytes clustering around epidermal Langerhans forming the characteristic Pautrier microabscesses (Girardi *et al*, 2004; Kim *et al*, 2005). Immature dendritic cells (Langerhans cells) in the epidermis may play an intimate role in the early pathogenesis of MF/SS by activating T cells through direct contact and providing the impetus for their clonal expansion (Berger *et al*, 2002). Progression of MF/SS is accompanied by clonal dominance of the malignant cells, (Vega *et al*, 2002) leading to the elaboration of Th2 cytokines, (Rook *et al*, 1997) impairment of the host's immune response, and further tumour cell growth (Girardi *et al*, 2004; Kim *et al*, 2005).

The survival of MF/SS patients varies markedly with the stage of disease at presentation, and although difficult to study, may in part be dependent on whether immunosuppressive therapy is administered (Table 1) (Kim and Hoppe, 1999; Kim *et al*, 1999; Zackheim *et al*, 1999). Patients in the early stages of MF are treated with topical agents and show a median survival not unlike that of matched control populations. Patients with advanced stages IVA-B defined by pathologically involved lymph nodes, blood, or visceral involvement have a poor prognosis, with a median survival of only 2.5 years in some studies (Kim and Hoppe, 1999). The appearance of large cell transformation within 2 years of diagnosis is also associated with a poor prognosis (Diamandidou *et al*, 1998).

## CURRENT TREATMENT OPTIONS

The primary goal of any therapy is to induce a long-term complete remission or cure. However, a less ambitious goal of decreasing the tumour burden, improving symptoms, and preserving quality of life, without further impacting the patient's already compromised immune system, is a more realistic situation. There are a limited number of therapeutic modalities, including skin-directed therapies, biologic-response modifiers, systemic agents, and experimental therapies, available to treat patients with MF/SS (Table 2). However, only extracorporeal photopheresis, denileukin diftitox, and bexarotene have FDA approval for this specific indication. In general, MF/SS is managed according to the stage of the disease (Table 3). Treatment decisions may be influenced by weighing the expected benefits against drug toxicities, patient tolerance and convenience, cost, and compliance with therapy.

Patients with early stages of MF have disease localised to the skin, where it can often be put into remission with skin-directed therapies. The usual progression of treatment begins with topical steroids or retinoid, followed by either topical chemotherapy with mustargen (mechlorethamine HCl) or with one form of phototherapy. Combinations of topical and phototherapy may hasten the time to response. Total skin electron beam irradiation is generally reserved for patients with generalised skin involvement, thick plaques, or tumours who have failed topical therapies. Aggressive chemotherapy cannot be justified for early MF because it does not prolong overall survival in comparison to conservative sequential therapy (Kaye *et al*, 1989).

In patients who fail to respond to skin-directed therapy or have more extensive lesions, the addition of biological response modifiers is the accepted therapeutic choice. As discussed in a previous detailed reviews of the literature, (Kempf *et al*, 2003; Zhang and Duvic, 2003) oral retinoids, either alone, or in combination with other skin-directed or biological response modifiers, represent a good low-risk treatment modality to control early MF. Retinoic acid receptor selective retinoids (isotretinoin and etretinate) are similar in efficacy with response rates of about 50% reported for each agent. Bexarotene oral capsules as monotherapy were able to induce responses in 54% of early (Duvic *et al*, 2001b) and 45% risk reduction of advanced, refractory CTCL at the optimised dose of 300 mg m^−2^ in two clinical Phase II trials (Duvic *et al*, 2001a, 2001b). Combined treatment modalities, including the retinoid isotretinoin, followed by TSEB (for stage I–II disease) or preceded by chemotherapy (for stage II and IV disease) (Duvic *et al*, 2003a) bexarotene plus PUVA, or photopheresis plus alpha interferon (IFN) and low-dose bexarotene, (Rook *et al*, 1999a) have been reported to give overall response rates of 82% in MF patients and 69% in SS patients (Kempf *et al*, 2003). Combinations of bexarotene with other modalities were most effective when one or two lipid-lowering agents and synthroid were simultaneously administered, giving response rates of over 70% (Talpur *et al*, 2002). Retinoids can be given orally, are active in 50% of the patients, and are a generally well tolerated first-line systemic therapy of CTCL; however, patients may fail to reach complete remission or may relapse while on treatment, requiring other approaches and therapies.

Another agent to show promise in treating CTCL is the tumour-targeted fusion protein, denileukin diftitox (Olsen *et al*, 2001). In patients with stage Ib to IVa CTCL who had received >3 prior therapies, denileukin diftitox therapy (9 or 18 *μ*g kg^−1^ day^−1^ for 5 days every 3 weeks for up to eight cycles) produced an overall response rate of 30% (20% partial response (PR), 10% complete response (CR)) and mediation duration of response of 6.9 months (range, 2.7 to >46.1 months). Pre-administration of low-dose corticosteroids with denileukin diftitox infusions blocked the commonly encountered infusion reactions and improved the response rate to 60% (Foss *et al*, 2001). Denileukin diftitox can produce a vascular leak syndrome (hypotension, hypoalbuminemia, and oedema), which can be partially prevented by saline infusions post-treatment. It can also induce a hypersensitivity reaction or transaminase elevations in some patients.

Patients with more advanced MF/SS or those who relapse and become resistant to initial treatments require a more aggressive therapeutic approach, which may involve biologic response modifiers, combination therapies, bone marrow transplantation, or chemotherapy. In addition to retinoids, both alpha and gamma IFNs are active biological response modifiers with immunostimulatory activity in CTCL. Alpha IFN combined with photopheresis and a retinoid are first line treatments for patients with SS (Gottlieb *et al*, 1996; Duvic *et al*, 2003b). Another biological response modifier, interleukin 12, has shown activity in three separate clinical trials and increases CD8+ T-cell infiltrates in CTCL lesions (Rook *et al*, 1999b).

Although combined chemotherapeutic regimens (e.g., CHOP (cyclophosphamide, vincristine, doxorubicin, prednisone), CMED (cyclophosphamide, methotrexate, etoposide, and dexamethasone), and ESHAP (etoposide, methyl prednisolone, high-dose cytarabine, and cisplatin)) have been utilised in treating CTCL and have response rates of about 60%, the duration of responses seen may be disappointingly brief. Combined chemotherapy often requires the placement of central lines, which in turn increase the risk of potentially fatal opportunistic infections in the setting of a compromised host immune system.

Nucleoside agents have recently been studied for treatment of advanced patients with CTCL. Several phase II studies concluded that gemcitabine has high response rates of 70% in patients with MF (Zinzani *et al*, 2000; Marchi *et al*, 2005). Gemcitabine is especially active against tumours (Duvic *et al*, 2006b) and is well tolerated, with minimal toxicity. Pentostatin has been studied as well and shows 60% response rates in patients with SS but is less active against tumours and nodal disease where the duration of response is only a few months (Kurzrock *et al*, 1999). Chemotherapy should be used for palliation in patients with nodal or visceral involvement or large cell transformation.

Bone marrow transplantation (autologous and allogeneic) has been employed to treat advanced CTCL but the overall clinical experience to date is limited to less than 20 patients. Autologous transplants have not cured the disease whereas non-ablative allogenic transplants show more promise in putting patients into complete remissions. Nevertheless, allogeneic transplantion was reported to produce remissions of 4.5 years, 15 months, and 9 months in three patients (Guitart *et al*, 2002). Further, the results of a retrospective study evaluating outcome in advanced CTCL patients population suggests that allogeneic haematopoietic stem-cell transplantation can induce durable clinical, molecular, and cytogenetic remissions in patients with advanced CTCL refractory to standard therapies (Molina *et al*, 2005).

It is clear from these studies that patients with refractory or transformed MF and with SS have a generally poor prognosis and therapy is typically palliative. There is an urgent need for new therapies, especially ones capable of reversing the defective apoptosis that is a characteristic of all CTCL cells. HDAC inhibitors such as vorinostat, may modulate the expression of genes controlling growth and differentiation of T-cell lymphomas and may represent an attractive new strategy for targeted therapy of CTCL.

## EVALUATION OF VORINOSTAT IN CTCL

### Preclinical studies

**Figure 1:**
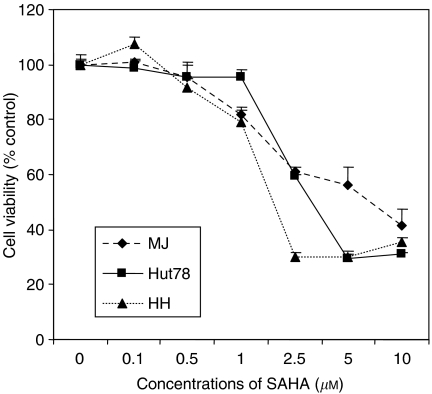
Vorinostat inhibited cell growth in three CTCL cell lines incubated *in vitro* for 24 or 48 h. Cells were aliquoted into 96-well plates and cell viability measured spectrophometrically at 490 nm after 4 h following addition of 3-(4,5-dimethylthiazol-2-yl)-5-(3-carboxymethoxyphenyl)-2-(4-sulfophenyl)-2*H*-tetrazolium inner salt. Reproduced with permission from Zhang C *et al*, *J Invest Dermatol* 2005 (Zhang *et al*, 2005).

Vorinostat demonstrates potent anti-tumour effects against three well-established CTCL cell lines (MJ [G11], Hut78, and HH) as well as in freshly isolated peripheral blood lymphocytes from 11 patients with a high percentage of circulating malignant T cells (Zhang *et al*, 2005). As shown in Figure 1, vorinostat inhibited cell growth of MJ, Hut78, and HH cell lines in a dose-dependent and time-dependent manner. For example, increasing the dose of vorinostat from 1 to 5 *μ*M increased growth inhibition of HH cells from 2 to 34% at 24 h and from 21 to 70% at 48 h. Analysis of the distribution of cells in various phases of the cell cycle using flow cytometry revealed that growth inhibition induced by vorinostat was associated with an increase in the percentage of cells in sub-G_1_ population and a loss of cells in the G_1_, S, and G_2_/M phases, suggesting vorinostat induces apoptosis (Zhang *et al*, 2005).

**Figure 2:**
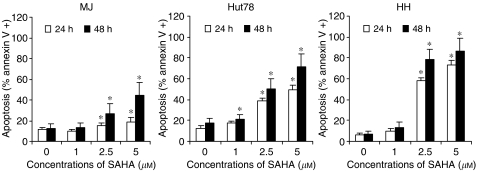
Effect of increasing doses of vorinostat on apoptosis assessed by annexin V binding in three CTCL cell lines after 24 or 48 h of incubation. Reproduced with permission from Zhang C *et al*, *J Invest Dermatol* 2005 (Zhang *et al*, 2005).

The proapoptotic effect of vorinostat was confirmed *in vitro* by flow cytometric analysis of annexin V protein binding to the cell surface of CTCL cell lines and freshly isolated peripheral blood lymphocytes from CTCL patients (Zhang *et al*, 2005). Annexin V binding is a novel technique to detect apoptosis and works by binding to phosphatidylserine residues at the outer plasma membrane. These residues become exposed as a result of loss of phospholipid asymmetry of the plasma membrane, an early event in apoptosis. Vorinostat increased the proportion of cells binding annexin V in all three CTCL cell lines (Figure 2) (Zhang *et al*, 2005). Importantly, vorinostat was associated with selective apoptosis, as evidenced by the ability of vorinostat to increase annexin V binding to freshly isolated peripheral blood lymphocytes obtained from SS/MF patients but to lymphocytes isolated from healthy donors.

### Mechanism of selective apoptotic action in CTCL cells

Selective apoptosis produced by vorinostat may potentially involve a variety of mechanisms, including an increase in p21^WAF1^, an alteration in bax/bcl-2 ratio, a decrease in Stat6 and phosphor-Stat6 proteins, and an activation of caspase-3 (Zhang *et al*, 2005). At this juncture, it seems unlikely that differences in the ability of vorinostat to inhibit HDAC activity or regulate the expression of key genes are the primary hypothetical mechanisms for differential sensitivity of tumour and normal cells to this agent. This assertion is based on the observations that the addition of vorinostat results in accumulation of acetylated histones in both normal fibroblasts from human lung and breast as well as transformed cells (WI-38, VA 13, and ARP-1), CTCL cell lines (MJ, Hut78, and HH), and peripheral blood lymphocytes from two patients with SS (Ungerstedt *et al*, 2005; Zhang *et al*, 2005). Nevertheless, the results presented by Ungerstedt *et al* (2005) do indicate that vorinostat may cause an accumulation of reactive oxygen species (ROS) and caspase activation in transformed but not normal cells as well as an increase in the level of Trx, a major reducing protein for many targets, in normal cells but not in transformed cells (Zhang *et al*, 2005).

Our *in vitro* studies (Zhang *et al*, 2005) of CTCL cell lines revealed that vorinostat at concentrations associated with apoptosis resulted in induction of expression of p21^WAF1^, a protein that has been shown to be responsible for cell cycle arrest and apoptosis induced by HDAC inhibitors (Mei *et al*, 2004; Somech *et al*, 2004). Although upregulation of p21^WAF1^ occurred in response to vorinostat, immunoblot analysis showed that this effect was independent of the tumour suppressor p53. In addition, vorinostat was found to impact the nuclear to cytoplasmic expression of p-Stat stat 3 proteins in CTCL cell lines (Duvic *et al*, 2006a). Stat proteins represent a family of transcription factors that, once activated, are believed to contribute to oncogenesis by stimulating cell proliferation and preventing apoptosis (Sommer *et al*, 2004; Turkson, 2004; Mitchell and John, 2005). Vorinostat at concentrations capable of producing apoptosis, decreased the expression of Stat6 and phosphor-Stat6 (but not Stat3 and phosphor-Stat3) in CTCL cell lines and peripheral blood lymphocytes from SS patients. Clearly, further research is needed to explore all of these mechanisms in greater detail.

### Phase I study

A recently completed phase I study of patients with advanced cancer described the effect of oral vorinostat in a patient with CTCL who had failed to respond to a total of five previous systemic therapies. Oral vorinostat administered at a dose of 200 mg twice daily for a total of 4 months resulted in a stabilisation of the disease. Although a second patient with peripheral T-cell lymphoma was enrolled, the report did not appear to describe the outcome of this patient. This finding is consistent with a recent phase I trial of another HDAC inhibitor (FR901228), which showed a PR in three patients with CTCL and one patient with peripheral T-cell lymphoma (Piekarz *et al*, 2001).

### Phase II study

In light of the favourable preclinical studies and the response of the CTCL patient to oral vorinostat in the phase I trial, a phase IIA clinical trial (Duvic *et al*, 2005) was conducted at our centre to determine the response rate, duration of response, pruritus relief, and safety profile of oral vorinostat in 33 patients with refractory or relapsed CTCL (stages IA-IVB) unresponsive to at least one conventional therapy. There were four patients who were treated twice in different dosing cohorts.

Patients were required to have adequate bone marrow, liver, and renal function, and an ECOG performance status of less than or equal to 2. Enrolled patients were treated with continuous daily dosing of oral vorinostat 400 mg, intermittent dosing of oral vorinostat 300 mg twice daily (for 3 days with 4 days rest), or oral vorinostat according to an induction/rest/continuous regimen (300 mg twice daily for 14 days, with 7 days rest followed by 200 mg twice daily). The primary efficacy end point of the study was the complete & partial response rate (CR, PR). However, the study also evaluated time to response, time to progressive disease, response duration, pruritus relief and safety. The response to therapy was categorised according to the World Health Organization criteria (Miller *et al*, 1981) as a CR, PR, stable disease, or progressive disease. The safety of vorinostat was assessed by utilising the National Cancer Institute Common Toxicity Criteria version 2.

Based on a preliminary analysis of intent-to-treat data, presented at the 2005 annual meeting of the American Society of Clinical Oncology (Duvic *et al*, 2005), a total of 10 out of 37 patients with heavily pretreated advanced refractory CTCL patients achieved a meaningful PR (>50% reduction in disease burden). Considering unique patients for an intent-to-treat analysis, eight out of 33 or 24% of the patients achieved a documented PR and there were no CRs (Duvic *et al*, 2006a).

The most common major toxicities that were possibly or probably related to oral vorinostat therapy were fatigue and gastrointestinal symptoms, including diarrhoea, altered taste, nausea, and dehydration from not eating/drinking. Thrombocytopenia was dose limiting in patients receiving oral vorinostat at the higher induction doses of 300 mg twice daily for 14 days. Overall, oral vorinostat at a dose of 400 mg daily provided the most favourable risk–benefit profile and, thus, this dose has been selected for a second Phase IIB trial currently ongoing at multiple centres.

The adverse effects associated with vorinostat therapy can be managed in several ways. Dehydration in elderly patients can be prevented by weighing patients at each visit, checking haemoglobin and creatinine levels and by using fruit juice, popsickles, smoothies, or sherbert to relieve a dry mouth and improve taste and appetite. In addition, nausea and vomiting can be controlled with ondansetron hydrochloride and diarrhoea treated with lomotil. The risk of haematologic side effects can also be minimised by avoiding aspirin and by monitoring for a rapid fall in platelet counts or a rapid change in international normalised ratio in those patients receiving coumadin.

## CONCLUSIONS

The HDAC inhibitor vorinostat increases apoptosis of CTCL cells *in vitro* at concentrations that are clinically relevant. Oral vorinostat has a rapid onset of action and exhibits significant clinical activity against transformed tumours, erythroderma, and nodes in heavily pretreated, refractory CTCL patients. Most patients with CTCL also experience significant itching relief with vorinostat therapy, and, hence, a marked improvement in their quality of life. Overall, vorinostat appears to be generally well tolerated, with fatigue and gastrointestinal symptoms being the most common side effects at lower doses and thrombocytopenia at higher doses. These side effects were dose related and reversible upon cessation of therapy. Additional studies are underway to define the exact mechanism (s) of by which vorinostat induces selective apoptosis in CTCL cells and to further evaluate the antitumour efficacy of vorinostat in a Phase IIb study in CTCL patients.

## Figures and Tables

**Table 1:** Prognosis of CTCL patients according to tumour stage at presentation

**Stage**	**Description**	**Survival**
1A	T1, patches and/or plaques involving <10% BSA with no palpable adenopathy	Similar to age-matched controls (Kim *et al*, 1999; Zackheim *et al*, 1999)
1B	T2, patches and/or plaque involving at ⩾10% BSA with no palpable adenopathy	⩾67% at 10 years (Kim *et al*, 1999; Zackheim *et al*, 1999)
IIA	T1/T2, patches and/or generalised plaque, clinically abnormal lymph nodes	
IIB	T3, cutaneous tumours	5 years (Kim and Hoppe, 1999)
III	T4, generalised erythroderma	
IVA-B	Pathologically involved lymph nodes or visceral involvement	2.5 years (Kim and Hoppe, 1999)

BSA=body surface area.

**Table 2:** Overview of current therapeutic options for MF/SS

*Skin-directed therapy*
• Topical corticosteroids (Farber *et al*, 1968; Zackheim *et al*, 1998)
• Topical chemotherapy (e.g., nitrogen mustard, (Vonderheid *et al*, 1989) carmustine (Zackheim *et al*, 1990))
• Topical retinoids (bexarotene, (Duvic *et al*, 2001a; Breneman *et al*, 2002) tazarotene (Apisarnthanarax *et al*, 2004))
• Topical imiquimod (Didona *et al*, 2004)
• Phototherapy (UVB, (Ramsay *et al*, 1992) NbUVB, (Gathers *et al*, 2002) PUVA (Herrmann *et al*, 1995))
• Electron beam therapy (Jones *et al*, 1995)

*Biological therapy*
• RXR retinoid (bexarotene) (Breneman *et al*, 2002; Duvic *et al*, 2001b)
• RAR retinoid (isotretinoin) (Neely *et al*, 1987)
• Interferons (Olsen, 2003)
• Granulocyte–macrophage colony-stimulating factor (Bouwhuis *et al*, 2002)
• Extracorporeal photopheresis (Duvic *et al*, 2003b)
• Fusion protein/toxin (denileukin diftitox) (Olsen *et al*, 2001)

*Other systemic therapies*
• Cytotoxic chemotherapy (methotrexate, (Zackheim *et al*, 2003) doxil,(Di Lorenzo *et al*, 2005) gemcitabine, (Sallah *et al*, 2001) etoposide, (Onozuka *et al*, 2004) pentostatin (Dearden *et al*, 2000))
• Bone marrow/stem cell transplantation (Soligo *et al*, 2003; Guitart *et al*, 2002)

*Experimental therapies*
• HDAC inhibitors (vorinostat, (Duvic *et al*, 2005) depsipeptide (Piekarz *et al*, 2001))
• Transimmunisation extracorporeal photopheresis (Girardi *et al*, 2002)
• Targeted monoclonal antibodies (CD52, (Lundin *et al*, 1998; Lundin *et al*, 2003) CCR4(Niwa *et al*, 2004)}, CD4 (McFarlane *et al*, 2005))
• Cytokines (IL-12, IL-2 (Rook *et al*, 2003))
• TLR agonists (CpG oligodeoxynucleotides) (Wysocka *et al*, 2004)
• Tumour vaccines (Seo *et al*, 2003)

UVB=ultraviolet B light, NbUVB=narrow-band ultraviolet B light, PUVA=psoralen plus ultraviolet A light, RXR=retinoid X receptor, RAR=retinoic acid receptor, TLR=toll-like receptor 9; IL=interleukin. Information extracted from Kim EJ *et al*, *J Clin Invest* 2005; **115**:798–812.

**Table 3:** Overview of initial and subsequent treatment options by stage for patients with MF/SS

**TNMB classification and staging**				
**IA**	**IB-IIA**	**IIB**	**IIIA,B**	**IVA,B**
*Initial therapy*				
Skin directed-therapies – topical chemotherapy PUVA UVB Local EBT	Topical chemotherapy Phototherapy TSEBT PUVA	Discrete tumours: – Local EBT+topical chemotherapy or PUVA Generalised tumours: – TSEBT+topical chemotherapy – PUVA+INF-*α* – PUVA+retinoids – Denileukin diftitox	ECPP PUVA Retinoids INF-*α* Methotrexate	Topical therapy+ – Palliative systemic chemotherapy – Combined systemic therapies (e.g., INF-*α*, retinoids, ECPP) – Local radiation for extracutaneous disease
*Subsequent therapy*				
	IFN-*α* Retinoids combined therapies – TSEBT/PUVA+topical chemotherapy – PUVA+INF-*α* – PUVA+retinoids – TSEBT+INF-*α*	Other combined therapies – TSEB+ECPP – Retinoids+INF-*α* – TSEBT+chemotherapy	Combined therapies: – ECPP+INF-*α* – PUVA+INF-*α* – PUVA+retinoids – Retinoids+INF-*α*	Bone marrow/stem cell transplant
*Salvge therapy*				
Newer/investigational therapies				

PUVA=oral psoralen-UV-A, EBT=electron beam therapy, TSEBT=total skin electron beam therapy, INF-*α*=interferon-*α*, ECPP=extracorporeal photopheresis

Information extracted from Kim EJ *et al* (Kim *et al*, 2005) and Kim YH and Hoppe RT (Kim and Hoppe, 1999).
